# The strategic breakdown: CHAC enzymes as regulators of glutathione homeostasis and disease implications

**DOI:** 10.3389/fmolb.2025.1724944

**Published:** 2025-12-18

**Authors:** Emma Schröder, Tida V. Jalilvand, Janina Kahl, Charlotte S. Kaiser, Eva Liebau

**Affiliations:** 1 Department of Molecular Physiology, Institute of Integrative Cell Biology and Physiology, University of Münster, Münster, Germany; 2 Department of Ophthalmology, Charité, Berlin, Germany

**Keywords:** CHAC1, CHAC2, glutathione, ferroptosis, cancer, BOTCH, endoplasmic reticulum stress

## Abstract

This review explores the critical function of the evolutionarily conserved ChaC-like enzyme family as central regulators of intracellular glutathione (GSH) homeostasis, focusing on the mammalian isoforms CHAC1 and CHAC2. We detail how these γ-glutamylcyclotransferases degrade GSH, thereby modulating cellular redox balance and integrating diverse stress signaling pathways. CHAC1 emerges as a key stress-responsive effector, transcriptionally upregulated *via* the ATF4-CHOP axis during endoplasmic reticulum stress and amino acid deprivation. Its role is especially crucial in the induction of ferroptosis, an iron-dependent cell death pathway, positioning it as a context-dependent modulator of cancer progression, neurotoxicity and neurodegeneration. Furthermore, we examine the opposing roles of CHAC1 and CHAC2 in stem cell fate decisions *via* NOTCH1 signaling and development. The complex duality of CHAC1 in oncology, acting as both a tumor suppressor by promoting ferroptosis and a potential oncogene in *TP53*-mutant backgrounds, alongside its functions in neuroprotection and immunity, underscores its therapeutic potential.

## 1 Introduction - glutathione and ChaC-like proteins

Glutathione (GSH), a tripeptide of γ-glutamylcysteinylglycine, is a key component of the thiol system and plays a vital role in maintaining cellular redox balance across compartments, tissues, and organisms. Its γ-glutamyl bond to the cysteine residue provides unique chemical properties and high stability within the cell, allowing GSH to function as a powerful redox buffer. The reducing capacity of GSH is essential for regulating the redox environment, as it can reversibly form oxidized glutathione (GSSG) through the coupling of two GSH molecules. This dynamic equilibrium enables precise control of the cellular redox state. The ratio of reduced to oxidized glutathione serves as a key indicator of the cellular redox potential, with even small changes in the GSH:GSSG ratio influencing protein function by altering cysteine oxidation and disulfide bond formation (Romero-Aristizabal et al., 2014). Additionally, the redox couple can scavenge radicals, as two glutathione thiyl radicals react to form GSSG (Deponte, 2022). Typically, the cytosol contains GSH in the millimolar range, while GSSG concentrations are usually in the nanomolar or lower range (Deponte, 2022). These levels are not static but instead vary across different compartments. These variations are influenced by multiple processes, including *de novo* synthesis, GSH degradation and metabolism, the uptake and export of GSH and GSSG and the efficient reduction of GSSG. Thus, cellular concentrations are tightly regulated by various mechanisms to maintain a stable GSH:GSSG ratio and redox potential.

In animals, GSH synthesis is regulated by feedback inhibition based on cysteine availability and the balance between the catalytic and regulatory subunits of glutamate-cysteine ligase (GCL) (Fraser et al., 2003; Lu, 2013; Majerus et al., 1971; Richman and Meister, 1975; Yang et al., 2002). Additionally, GSH can be regenerated from GSSG by glutathione reductases (GR). However, many organisms can survive without these enzymes and some organisms completely lack GR, suggesting that alternative mechanisms for regenerating GSSG may exist (Buchholz et al., 2010; Kanzok et al., 2001; Marty et al., 2009; Tan et al., 2010).

**FIGURE 1:**
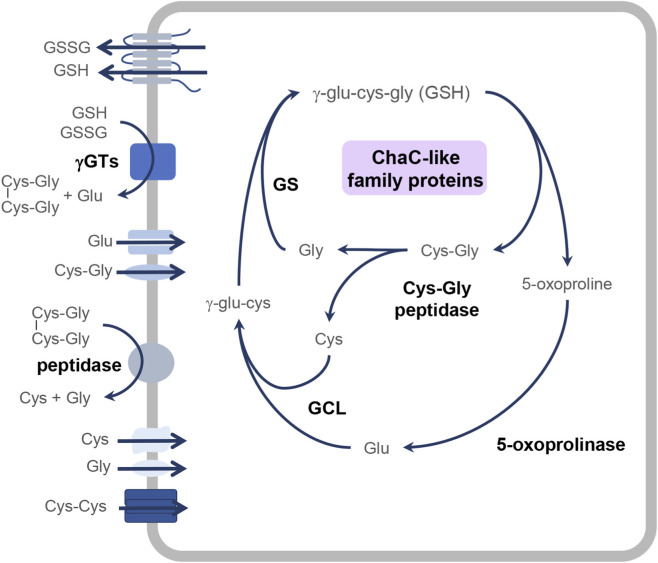
The novel glutathione cycle of intracellular GSH biosynthesis and turnover. Intracellular glutathione biosynthesis from glutamate, cysteine and glycine occurs through a two-step process mediated by the enzymes glutamate-cysteine ligase (GCL) and glutathione synthetase (GS). The degradation of GSH is mediated by ChaC-like family proteins, resulting in the formation of 5-oxoproline and cysteinylglycine. These products are further broken down by 5-oxoprolinases and Cys-Gly peptidases and the resulting glutamate, cysteine, and glycine can be reused for GSH biosynthesis. Following efflux from the cell, oxidized glutathione can be salvaged by γ-glutamyltransferases (γGT). The glutamate and cysteinylglycine or bis-cysteinylglycine formed in this reaction are degraded by peptidases to cysteine and glycine, which are then transported back into the cell.

While synthesis and regeneration have been well characterized, GSH degradation remained less clear. In 1970, Orlowski and Meister proposed the γ-glutamyl cycle, which includes the synthesis, breakdown, export and import of GSH as a key process for amino acid uptake (Orlowski and Meister, 1970). This model was critically reassessed a few years ago by Bachhawat and Yadav, who extended it to include the intracellular degradation of GSH (Bachhawat and Yadav, 2018) (Figure 1).

Initially, the degradation of extracellular GSH by membrane-bound γ-glutamyltransferases (γGTs) was considered the major pathway for GSH breakdown (Orlowski and Meister, 1970). Today, however, it is increasingly recognized that these enzymes play only a complementary role. While γGTs initiate extracellular GSH breakdown, the primary intracellular degradation pathway is now thought to be mediated by the cationic transport regulator homolog (ChaC)-like family of γ-glutamylcyclotransferases (γGCT) (Bachhawat and Yadav, 2018).

**FIGURE 2:**
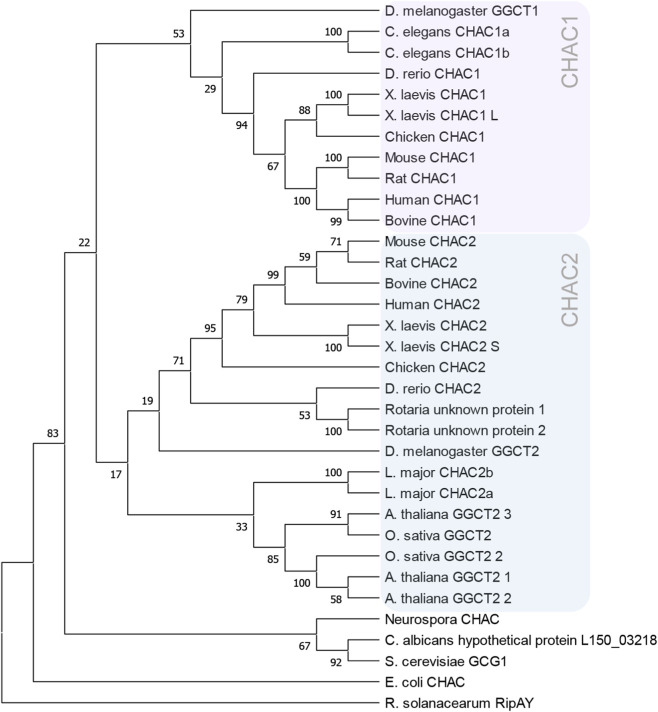
Phylogenetic overview of ChaC-like proteins. Included are canonical model organisms as well as diverse species representing major evolutionary lineages, from microbes to vertebrates. The neighbor-joining tree was constructed using MEGA11 software and MUSCLE algorithm with 100 bootstrap method. The violet box highlights all CHAC1 enzymes, while the blue box highlights all CHAC2 enzymes.

ChaC-like family proteins are remarkably conserved, being found across all phyla from bacteria to humans, where they catalyze intracellular GSH degradation and yield 5-oxoproline and cysteinylglycine (Figure 2). The cytosolic CHAC proteins have a γGCT-fold, which places them within the γGCT protein family (Kumar et al., 2012; Mungrue et al., 2009). In both mice and humans, two homologs have been identified: CHAC1 and CHAC2. While CHAC1 is limited to higher eukaryotes, CHAC2 is present across a broad phylogenetic range. Accordingly, plants, bacteria and lower eukaryotes express only CHAC2, while higher eukaryotes also express CHAC1 (Kaur et al., 2017). CHAC1 demonstrates higher catalytic efficiency and is upregulated during development or in response to stress. In contrast, CHAC2 possesses lower efficiency and was initially thought to be constitutively expressed (Kaur et al., 2017). Yet, this view has shifted with recent findings that uncovered a developmental expression pattern for CHAC2 and a role in stem cell fate determination (Wang et al., 2017).

Beyond CHAC1 and CHAC2, additional ChaC-like enzymes broaden the functional spectrum of this protein family. Bioinformatic analyses have further categorized the ChaC-like protein family into classes I and II, following the discovery of the thioredoxin-dependent ChaC-like protein RipAY in the phytopathogens *Ralstonia solanacearum* and *Acidovorax citrulli.* RipAY is a class II enzyme that is found exclusively in these two plant pathogens (Fujiwara et al., 2016).

Since its initial identification as a potential regulator of the *Escherichia coli cha* (Ca^2+^/H^+^ antiporter) operon, which is involved in cation transport (Ivey et al., 1993), the ChaC protein family has emerged as a critical regulator of diverse biological processes. In mammalian cells, *CHAC1* was identified as an endoplasmic reticulum (ER) stress-responsive gene, which prompted detailed investigations into its molecular structure and functional mechanisms (Kumar et al., 2012; Mungrue et al., 2009). Recent studies have elucidated the proapoptotic functions of CHAC proteins and their involvement in ferroptosis, cancer progression and neurodegenerative diseases (Goebel et al., 2012; Kumar et al., 2012; Zhou, Q. et al., 2024). The following sections explore these aspects in more detail. We focus on their molecular mechanisms in GSH metabolism, their roles in ER stress and ferroptosis, their dual functions in cancer and their impact on stem cell dynamics and neurodegenerative pathways.

### 1.1 Molecular and enzymatic properties of mammalian CHAC enzymes

In humans, the *CHAC1* gene encodes six transcripts, which give rise to three different isoforms: isoform A, isoform B and isoform X1. Due to a large 44-base pair deletion, isoform B is non-functional, while isoforms A and X1 exhibit enzymatic activity. These functional isoforms differ in their translation start site, resulting in length variations, with isoform A consisting of 222 amino acids and isoform X1 containing 264 amino acids, including an additional 42 amino acid segment at the N-terminus. Although isoform X1 is absent in certain higher organisms, such as mice, elephants, dogs and chickens, it is highly conserved across many other species (Kaur et al., 2017). The primary and most prevalent isoform, however, is the shorter isoform A (Crawford et al., 2015). CHAC1 is the name used throughout this review when discussing the protein’s enzymatic function in glutathione degradation during stress responses, ferroptosis and disease, whereas the name CHAC1/BOTCH is used when emphasizing its specific role in development and stem cell fate *via* the inhibition of NOTCH1 signaling.

ChaC-like proteins are generally characterized by their highly conserved γGCT domain, which enables them to degrade γ-glutamyl amino acids. A distinct feature of these proteins is their structural fold, which includes α-helices surrounding a β-barrel structure and an overlapping β-strand motif. This BtrG/γGCT fold distinguishes these enzymes from others exhibiting cyclotransferase-like activity (Oakley et al., 2008). All γGCT enzymes possess a highly conserved pocket, proposed to be the active site, which contains a catalytically relevant glutamate residue. Additionally, a short loop connected to the β1 strand contains backbone amide nitrogen atoms critical for substrate binding. The loops that surround the active site are poorly conserved, allowing for functional diversity and substrate specificity (Oakley et al., 2008).

Bioinformatic analysis has identified a diverse set of residues that are critical for CHAC1 activity and collectively form its active site (Suyal et al., 2023). Key residues ^38^YGSL^41^, D^68^, R^72^, E^115^ and Y^143^ are responsible for high affinity substrate binding *via* salt bridge interaction with the glutamate (E^115^), hydrogen bonding as well as a cation π-interaction involving Y^143^. The polar and charged residues lining the enzyme´s binding pocket likely contribute to the formation of an electrostatic environment essential for substrate interaction (Suyal et al., 2023). Notably, mutation of E^115^ inactivates CHAC1, preventing GSH depletion *in vivo* (Kumar et al., 2012).

**FIGURE 3:**
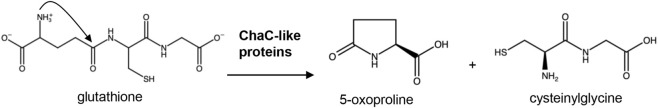
Schematic overview showing the degradation of glutathione mediated by ChaC-like proteins.

The molecular mechanism of γ-glutamyl amino acid degradation by γGCT family enzymes is well understood. The catalytic glutamate residue E^115^ accepts a proton from the γ-glutamyl group of the substrate, initiating a nucleophilic attack by the amine on the amide carbon atom. This results in the formation of an oxyanion intermediate before forming 5-oxoproline. Finally, the protonated glutamate residue donates a hydrogen to the amine of the γ-linked peptide, releasing an amino acid with a free amino group (Figure 3) (Oakley et al., 2008).

The ChaC-like family proteins, including CHAC1, preferentially degrade reduced glutathione but can also break down other γ-glutamyl amino acids (Chi et al., 2014; Kumar et al., 2012). During characterization of the enzyme, Kumar et al. demonstrated that CHAC1 degrades reduced glutathione and, to a lesser extent, γ-glutamyl-alanine (Kumar et al., 2012). Additionally, Chi et al. identified its activity towards γ-glutamyl-alanine and γ-glutamyl-glycine. This allows CHAC1 to post-translationally regulate protein activity by binding and cleaving specific γ-glutamyl amino acids (Chi et al., 2014).

Although CHAC2 enzymes in *Leishmania major* were shown to degrade only reduced and not oxidized glutathione, this specificity has yet to be confirmed for CHAC1 (Das et al., 2022). If confirmed, this selectivity of CHAC1 for GSH could significantly alter the GSH:GSSG ratio, thereby influencing the cellular redox potential.

CHAC2 is both evolutionarily older and structurally distinct from CHAC1. The human CHAC2 protein predates CHAC1, with the two sharing only 50% sequence identity (Kaur et al., 2017). The human CHAC2 protein consists of 184 amino acids, has a molecular weight of approximately 20.9 kDa, and is encoded on chromosome 2p16.2 (Chand et al., 2022; Kaur et al., 2017; Nguyen et al., 2020). Structurally, CHAC2 typically forms a dimer. A key feature is its flexible loop 2, which may serve a gating function for substrate binding specificity. The residues of the key ^6^YGS^8^ motif within the γGCT fold are located in loop 1, while the unique dimerization loop 2 is flipped away from the enzyme’s core domain (Nguyen et al., 2020). The dimer interface is formed by the interaction between loop 2 of one monomer and loop 1 of the other, stabilized by hydrogen bonds and salt bridges, resulting in a more open and flexible active site pocket compared to other γGCTs. Notably, CHAC2 displays significant structural differences in the loops surrounding its active site when compared to other γGCTs. For instance, where enzymes like CHAC1 exhibit a helical conformation in this region, CHAC2 has a flexible loop. Mutation of E^74^ to glutamine (Q) also induced helical formation in the CHAC2 loop 2, suggesting it might be the catalytically relevant glutamate residue that alternates with E^83^ (Nguyen et al., 2020).

## 2 CHAC1 as a central integrator of glutathione homeostasis, stress signaling and ferroptosis

Glutathione homeostasis is essential for cellular and organismal health due to its central role in maintaining redox balance. Mammalian CHAC1, with its unique activity, has emerged as a critical regulator of diverse biological processes. Initially identified as a stress-responsive protein upregulated during the unfolded protein response (UPR) and ER stress (Mungrue et al., 2009), CHAC1 plays dual roles in cellular regulation. Its primary function involves degrading reduced GSH, thereby disrupting GSH redox balance and shifting the cell towards a more oxidized state. This oxidative shift influences pathways that are sensitive to redox changes. These include ER stress signaling, the integrated stress response (ISR) and reactive oxygen species (ROS)-mediated apoptosis. It also modifies proteins by altering their thiol groups, which can change how these proteins function and regulate cellular activities. Furthermore, CHAC1 negatively regulates the NOTCH1 receptor, a key player in determining cell fate, particularly in neuronal stem cells, affecting their development and differentiation (Chi et al., 2012).

### 2.1 CHAC1 upregulation under arsenite stress and amino acid depletion

As a stress-responsive protein, CHAC1 is critically involved in the cellular stress response. Its expression is induced by arsenite (AsIII), amino acid depletion, and the pollutant perfluorooctane sulfonate (Hamano et al., 2020; Miljkovic et al., 2023; Örd et al., 2016; Qiu et al., 2025; Sumi et al., 2023; Yang et al., 2025).

During arsenite stress, CHAC1 promotes apoptosis in HaCaT cells by degrading GSH and increasing oxidative stress sensitivity (Sumi et al., 2023). Interestingly, a decrease in CHAC1 levels enhances cell survival and stress tolerance to As^III^, with CHAC1 knockdown reducing sensitivity to H_2_O_2_. In mouse embryonic fibroblasts, the elevation of CHAC1 protein expression following As^III^ exposure is negatively regulated by the Tribbles homolog 3 (TRIB3), which limits CHAC1′s pro-death potential (Örd et al., 2016). Furthermore, CHAC1 induction is directly suppressed by m^6^A methylation. METTL3 deposits m^6^A marks on *CHAC1* transcripts, leading to their recognition by YTHDF2 and subsequent degradation. Under arsenite stress, this METTL3/YTHDF2 interaction is inhibited, which stabilizes *CHAC1* mRNA and increases its expression (Qiu et al., 2025).

In addition to arsenite-induced expression, the *CHAC1* gene is transcriptionally activated during cysteine, serine or arginine depletion (Chen et al., 2017; Hamano et al., 2020; Miljkovic et al., 2023; Ward et al., 2024). The serine deprivation-dependent induction is regulated by the activating transcription factor 4 (ATF4) and can be reversed by the addition of glycine and serine, but not by other amino acids like leucine or cysteine (Hamano et al., 2020). The authors suggest that in HePa1-6 cells, CHAC1 expression is suppressed by serine synthesized from glycine (Hamano et al., 2020).

### 2.2 CHAC1 regulation through the ATF4-CHOP axis during ER stress

The link between amino acid depletion and ATF4 directly connects to a major upstream regulator of CHAC1: the ER stress pathway. The PERK-ATF4-CHOP pathway is a central signaling cascade activated in response to ER stress, serving as the cell´s primary reaction to dysfunction and playing a crucial role in cellular survival. However, if ER stress remains unresolved, it can lead to apoptosis, making this pathway particularly relevant in the context of neurodegenerative diseases. In addition to the PERK-ATF4-CHOP axis, other key ER stress pathways, such as IRE1/XBP1 and ATF6 also contribute to the UPR, collectively managing protein misfolding and restoring homeostasis, or, if stress persists, trigger cell death (Tabas and Ron, 2011).

**FIGURE 4:**
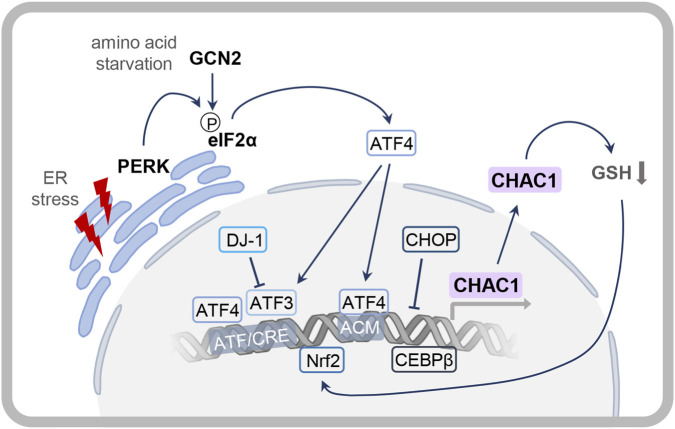
ER stress-dependent regulation of the *CHAC1* gene. In response to ER stress, CHAC1 expression is regulated downstream of the PERK-ATF4-CHOP pathway. ER stress induces the UPR pathways through induction of PERK resulting in elevated levels of phosphorylated eIF2α and translation of ATF4. The transcription factor ATF4 binds the promoter region of *CHAC1* at the ATF/CRE and ACM motif inducing *CHAC1* transcription. Additionally, the transcription factors ATF3, NRF2 and CEBPβ are involved in increased *CHAC1* gene expression, while DJ-1 and CHOP have an indirect inhibitory effect. In response to amino acid starvation, the kinase GCN2 is responsible for phosphorylation of eIF2α leading to increased *CHAC1* expression. High CHAC1 levels result in lower GSH content leading to increased NRF2 activation and a positive feedback loop in NRF2-mediated *CHAC1* expression.

Various studies targeting ER stress signaling pathways and components of the UPR have demonstrated that *CHAC1* expression is upregulated downstream of the ATF4-dependent signaling pathway (Figure 4). Additionally, stress-inducing agents such as tunicamycin, thapsigargin and histidinol have been shown to modulate CHAC1 regulation, indicating its involvement not only during ER stress but also in the context of the ISR (Crawford et al., 2015). At the mechanistic level, the binding of ATF4 to the *CHAC1* promoter is the primary mechanism for the transcriptional upregulation of the gene. While nuclear factor erythroid 2-related factor 2 (NRF2) and CCAAT/Enhancer Binding Protein γ (CEBPγ) also contribute to a minor increase in *CHAC1* transcription, their roles are less pronounced compared to ATF4 (Crawford et al., 2015).

ATF4 and ATF3 regulate *CHAC1* expression by binding to specific sites within its promoter, the ATF/cAMP response element (CRE) at position −267 and the ATF/CRE modifier (ACM) motif at −248. ATF3 primarily regulates both baseline and stress-induced *CHAC1* expression by binding to the ATF/CRE site. In contrast, ATF4 mainly drives *CHAC1* transcription under stress conditions through its interaction with both the ATF/CRE and ACM motif (Crawford et al., 2015; Nomura et al., 2020).

In addition to ATF4, CHOP plays a critical role in regulating CHAC1 during UFR (Crawford et al., 2015; Mungrue et al., 2009). While both are induced by ER stress and promote cell death, they exert opposing effects on the *CHAC1* promoter. ATF4 directly binds to the ATF/CRE element to activate transcription. In contrast, CHOP, which is itself transcriptionally upregulated by ATF4, acts as a negative feedback regulator. It significantly represses CHAC1 promoter activity without binding DNA directly. Instead, CHOP appears to act as a transcriptional decoy. It likely sequesters key binding partners such as ATF4 and C/EBPβ, preventing them from activating the *CHAC1* gene (Nomura et al., 2020). This sophisticated crosstalk, where parallel pro-apoptotic pathways (ATF4-driven induction and CHOP-mediated fine-tuning) converge on the *CHAC1* promoter, allows for precise control of cell fate under prolonged ER stress (Nomura et al., 2020).

The conservation of this regulatory axis is highlighted in other models. In *Caenorhabditis elegans*, F22F7.7 encodes an ortholog of human CHAC1. Research by Statzer et al. demonstrated that this ortholog (CHAC-1) is regulated by both the transcription factor ATF-4 and the mTORC1 pathway, linking it functionally to the reverse transsulfuration pathway. Specifically, ATF-4 enhances stress resistance and promotes longevity in worms by upregulating the transsulfuration enzyme CTH-2, increasing hydrogen sulfide (H_2_S) production (Statzer et al., 2022).

In mammalian cells, stress conditions lead to the upregulation of *CHAC1* through the action of ATF4. CHAC1 degrades GSH, leading to GSH depletion and oxidative stress. This oxidative imbalance might stabilize and activate NRF2 by disrupting its KEAP1-mediated degradation (Kreß et al., 2023). Furthermore, evidence indicates that NRF2 can transcriptionally upregulate CHAC1, as observed in ferroptosis-resistant melanoma cells where CHAC1 induction occurs independently of ER stress (Gagliardi et al., 2019) and modestly at the promoter level (Crawford et al., 2015) The stabilized NRF2 enhances expression of antioxidant defense genes, including the cystine/glutamate antiporter system (xCT) (subunit SLC7A11), which imports cystine for *de novo* GSH synthesis (Kreß et al., 2023). This creates an integrated feedback mechanism: CHAC1 provides GSH-derived glutamate to support the SLC7A11 antiporter and it promotes NRF2 activation. Consequently, CHAC1 serves as a central mediator connecting ISR (ATF4) to the antioxidant response (NRF2) and metabolic adaptation (SLC7A11), thereby enabling cells to dynamically fine-tune redox homeostasis and maintain metabolic flexibility during stress (Kreß et al., 2023).

The *PARK7* gene, which encodes the DJ-1 protein, is a causative gene for autosomal recessive early-onset Parkinson’s disease (Clements et al., 2006). Recent studies highlight its role in cellular stress pathways. Specifically, DJ-1 was found to negatively regulate ATF3, which in turn reduces CHAC1 expression thereby limiting GSH degradation (Clements et al., 2006; Ge et al., 2022). Another study shows DJ-1 and ATF3 to cooperatively prevent degradation of NRF2, further linking DJ-1 to oxidative stress modulation (Amatullah et al., 2021).

### 2.3 CHAC1 and ferroptosis

Programmed cell death is an actively regulated process, governed by a complex network of genes and proteins, that is critical for normal physiological functions across organisms (La Marca et al., 2024). Ferroptosis, an iron-dependent form of programmed cell death first identified by Dixon et al., is characterized by excessive intracellular iron accumulation, lipid peroxidation and distinct biochemical and morphological features (Dixon et al., 2012). Biochemically, ferroptosis involves suppression of the cystine/glutamate antiporter system xCT and decreased glutathione peroxidase 4 (GPX4) activity. This impairment of the cellular antioxidant machinery leads to ROS accumulation and mitochondrial damage, such as reduced volume and cristae breakdown (Liu et al., 2021). Mechanistically, ferroptosis is distinct from other forms of programmed cell death: unlike apoptosis or pyroptosis, it does not depend on caspase activation, nor is it primarily driven by ATP depletion or mitochondrial ROS generation, which are central to necroptosis.

In mammals, ferroptosis has been implicated in diverse pathologies, including neurodegenerative diseases, stroke, traumatic brain injury and kidney degeneration, as well as in carcinogenesis, where it may exert a tumor-suppressor function. Although the physiological role of ferroptosis remains unclear, it can be triggered by pharmacological disruption of cellular lipid peroxidation repair or by oxidative stress associated with degeneration. Consequently, inducing ferroptosis has gained attention as a potential therapeutic approach in cancer research (Stockwell et al., 2017).

During ferroptosis, lipid radical formation is induced by the accumulation of ROS leading to plasma membrane lipid peroxides (Jiang et al., 2021). To counteract this lipid peroxidation, cells rely on antioxidant defenses centered around GPX4 and NADPH-ferroptosis suppressor protein 1 (FSP1). Together with other lipid-soluble antioxidants, these pathways suppress membrane lipid peroxidation and preserve membrane integrity, thereby preventing ferroptotic cell death (Bersuker et al., 2019; Doll et al., 2019; Ingold et al., 2018; Yang et al., 2014).

**FIGURE 5:**
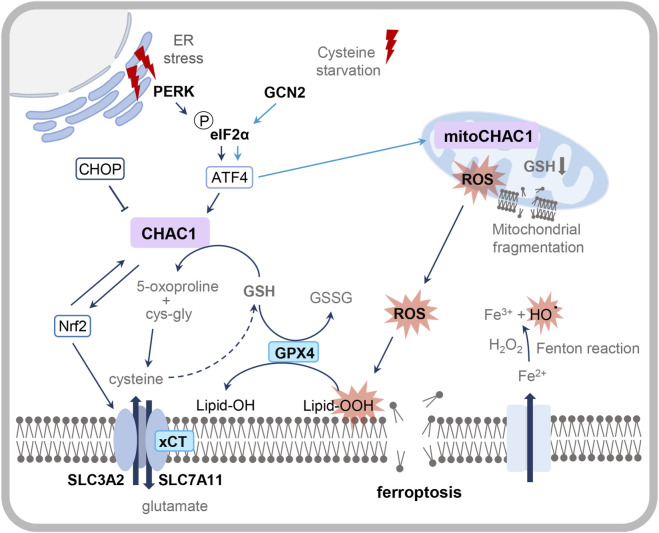
The ER stress-mediated ATF4-CHOP-CHAC1 axis in ferroptosis induction. During ferroptosis, ER stress or cysteine starvation activate the ATF4-CHOP-CHAC1 cascade resulting in elevated CHAC1 levels. Increased intracellular iron levels lead to enhanced ROS accumulation through the Fenton reaction with concomitant lipid peroxidation and cell death. CHAC1 promotes ferroptosis by degradation of GSH, which impairs GPX4 activity and accelerates lipid peroxidation. The transcription factor NRF2 regulates CHAC1 activity in a feedback loop while simultaneously activating xCT-mediated cystine uptake and counteracting intracellular cysteine deprivation. During cysteine starvation-induced ferroptosis, a fraction of CHAC1 localizes to the mitochondria to ensure respiratory function (light-blue arrows). By increasing cysteine levels through GSH degradation, Fe-S cluster protein synthesis is maintained. This leads to higher lipid peroxidation and mitochondrial fragmentation. The enzyme GPX4 and transporter system xCT (blue boxes) of the antioxidant system counteract ferroptosis. The combined loss of GPX4 activity and mitochondrial dysfunction synergistically amplify oxidative damage, positioning CHAC1 as a central integrator of ER stress signaling and ferroptotic cell death.

Ferroptosis can be triggered by various factors, with cysteine deprivation being one of the primary inducers. In cancer cells, cysteine deficiency blocks GSH synthesis and, consequently, the activity of GPX4 and its capacity to suppress ferroptosis is impaired. Additionally, cysteine starvation also activates the amino-acid starvation branch of the ISR, specifically through the GCN2-eIF2α-ATF4 axis. GCN2 phosphorylates eIF2α, leading to ATF4 induction and upregulation of downstream targets including *CHAC1*. CHAC1 further exacerbates cellular stress by degrading GSH, intensifying oxidative damage and ultimately driving ferroptotic cell death (Figure 5) (Chen et al., 2017).

While the eIF2αK3/PERK-eIF2α-ATF4 cascade, another branch of the ISR, can modulate ferroptosis in the context of ER stress, cysteine deprivation selectively activates GCN2 rather than PERK, highlighting distinct regulatory mechanisms (Chen et al., 2017).

Cysteine starvation triggers mitochondrial fragmentation, impairs mitochondrial function and leads to the accumulation of ROS (Chen et al., 2017). To counteract this stress, *CHAC1* is upregulated through the ATF4-dependent ISR pathway, where it degrades GSH to liberate cysteine needed for the synthesis of Fe-S cluster proteins (Ward et al., 2024). Particularly, in non-small lung cancer cells this CHAC1-mediated supply of cysteine is essential for sustaining the high oxidative metabolism and demanding energy household (Ward et al., 2024). Under cysteine deprivation, *CHAC1* is the most strongly upregulated gene, primarily *via* the ATF4-dependent ISR pathway. In contrast, its paralog *CHAC2* remains unresponsive, highlighting CHAC1’s unique role in stress adaptation (Ward et al., 2024). Interestingly, despite lacking a canonical mitochondrial targeting sequence, a small pool of CHAC1 localizes to mitochondria. This mitochondrial fraction is functionally critical, as demonstrated by the restoration of ferroptosis sensitivity in CHAC1-null cells through expression of a mitochondrially targeted CHAC1 (Ward et al., 2024).

Under cysteine deprivation, the ATF4-dependent ISR upregulates *CHAC1* to sustain mitochondrial respiration *via* GSH catabolism. CHAC1-mediated GSH degradation in the matrix supports Fe–S cluster proteins and electron transport (Ward et al., 2024). However, this preservation of mitochondrial function creates a paradox: by maintaining the electron transport chain, CHAC1 inadvertently supplies the ROS that propagate iron-dependent lipid peroxidation (Gao et al., 2019). Therefore, CHAC1 drives ferroptosis *via* two convergent mechanisms: the direct depletion of glutathione and the indirect facilitation of mitochondrial ROS production, thereby coupling sustained metabolic activity to ferroptotic cell death under cysteine limitation.

Ferroptosis is initiated by iron-dependent lipid peroxidation, marked by the accumulation of ROS, malondialdehyde (MDA) buildup and iron overload. This process is driven by disrupted GSH homeostasis, resulting from diminished GSH synthesis and impaired activity of two critical antioxidant systems: (1) the cystine/glutamate antiporter xCT, which imports cystine to fuel GSH production and (2) GPX4, which utilizes GSH to convert lipid peroxides into less toxic lipid alcohols (Cao and Dixon, 2016). Inhibition of xCT by agents such as erastin, lowers GSH synthesis and impairs GPX4 activity, thereby exacerbating lipid peroxidation (Cao and Dixon, 2016; Stockwell et al., 2017; Yang et al., 2014). CHAC1 amplifies this oxidative cascade by degrading residual GSH, further elevating ROS levels and preventing GPX4-mediated detoxification. The resulting suppression of GPX4 creates a self-reinforcing cycle, accelerating ROS accumulation and lipid radical formation. Consequently, the balance between xCT/GPX4 activity and ROS/iron/MDA levels determines cellular fate, with dysregulation tipping the scale toward ferroptotic cell death.

By degrading GSH, CHAC1 perturbs redox homeostasis and can effectively blunt the NRF2-driven cytoprotective response. *CHAC1* upregulation has been shown to be associated with the suppression of SLC7A11 and NRF2 and a surge in lipid peroxidation. For example, Yu et al. (2024) showed that treating bladder cancer cells with the anti-tumor compound brusatol boosted CHAC1 expression while simultaneously lowering SLC7A11 and NRF2 levels (Yu et al., 2024). This supports a model in which CHAC1-mediated GSH depletion destabilizes NRF2 and reduces cystine import, allowing iron-dependent lipid peroxides to accumulate and trigger ferroptosis. Yet, under regulation of the ISR pathway an ATF4-mediated upregulation of *NRF2 via* CHAC1 activity was shown as well (Kreß et al., 2023), revealing a context-dependent interplay between CHAC1 and NRF2 that merits further study.

#### 2.3.1 The dual role of CHAC1 in cancer

CHAC1’s function in cancer is complex and highly context-dependent. Cancer cells exhibit heightened metabolic activity, resulting in elevated ROS levels compared to normal tissues, which drives persistent oxidative stress (Moloney and Cotter, 2018). To counteract this imbalance, cancer cells adapt by upregulating GSH biosynthesis, maintaining a redox equilibrium that supports survival and proliferation (Traverso et al., 2013).

Within this context, CHAC1 has emerged as a molecule exhibiting a dual role in cancer biology. While its expression pattern varies across tumor types, *CHAC1* is frequently upregulated in aggressive malignancies, such as breast and ovarian cancer, where it correlates with advanced tumor differentiation and potentially serves as a prognostic marker. For instance, elevated CHAC1 mRNA levels are associated with increased recurrence risk in these cancers, underscoring its clinical relevance (Goebel et al., 2012). Yet, CHAC1 also exhibits proapoptotic activity in some cancer models, highlighting its potential as a therapeutic target (Joo et al., 2012).

Pan-cancer analyses reveal that *CHAC1* expression is highly variable, being upregulated in many cancers (e.g., breast, ovarian, lung, bladder carcinomas) and downregulated in others (e.g., certain head/neck, kidney, and brain cancers) (Li, D. et al., 2021; Sun et al., 2024; Zhang, T. et al., 2024). A comprehensive summary of CHAC1 expression across tumor types, along with TP53 status and ferroptosis susceptibility, is provided in Supplementary Table S1.

Elevated CHAC1 expression in LUAD correlates with aggressive tumor progression and poor patient prognosis (Pan et al., 2024). This association has been consistently replicated in studies of uveal melanoma, breast, and ovarian cancers, where *CHAC1* upregulation marks advanced disease states (Goebel et al., 2012; Liu, Y. et al., 2019). Similarly, *CHAC1* upregulation in renal cell carcinoma (RCC) is linked to advanced tumor stage and higher histological grade, suggesting its role in cancer progression and malignancy (Li, D. et al., 2021). Mechanistically, the increase in CHAC1 expression in cancer cells can be linked to the induction of ferroptosis through the ATF4 mediated UPR branch (Wang et al., 2021).

The elevated CHAC1 expression in LUAD promotes tumor proliferation and metastasis by enhancing glycolysis, lactate production, and ATP generation through regulation of pyruvate kinase M2 (PKM2). Mechanistically, E2F1-mediated *CHAC1* upregulation promotes PKM2 SUMOylation enabling PKM2 nuclear translocation or kinase activation (Pan et al., 2024). This, in turn, activates STAT3 signaling, leading to the upregulation of glycolysis-associated genes, notably independent of CHAC1’s established role in ferroptosis induction (Pan et al., 2024).

CHAC1’s contribution to tumor progression is markedly influenced by the cellular and tumor context, differing across various cancer types. In breast cancer, CHAC1 overexpression promotes both proliferation and migration, while in ovarian cancer, its impact is primarily restricted to cell migration, with minimal effect on proliferation (Goebel et al., 2012). Interestingly, Metha et al. (2022) found that CHAC1 overexpression in various breast cancer cells is often driven by hypomethylation of its promoter region, particularly in tumors harboring mutant TP53 (Mehta et al., 2022). Additionally, cancer cells carrying a mutant TP53 protein exhibit increased CHAC1 levels and show greater resistance to ferroptosis (Leu et al., 2019; Mehta et al., 2022).

TP53, encoded by the *TP53* gene and mutated in over 50% of human cancers, is essential for cell-cycle regulation and the induction of programmed cell death; tumors harboring mutations in the tumor suppressor therefore exhibit high grade invasiveness and metastatic potential (Duffy et al., 2018; Silwal-Pandit et al., 2017). In *Helicobacter pylori*-driven gastric carcinogenesis, infection sharply upregulates *CHAC1*. This upregulation exhausts intracellular GSH, leading to ROS buildup and oxidative DNA damage that can ultimately generate *TP53* mutations, a process that is halted when CHAC1 is knocked down (Wada et al., 2018).

TP53 induces ferroptosis primarily by transcriptionally repressing the expression of SLC7A11 (Le et al., 2015; Liu and Gu, 2022). This repression limits cystine uptake, depleting GSH and promoting ferroptotic cell death. Consequently, mutations in *TP53* lead to a loss of this repression, resulting in sustained SLC7A11 activity and greater cellular resistance to ferroptosis (Leu et al., 2019). Notably, the acetylation-defective mutant p53^3^KR, which cannot induce cell-cycle arrest, senescence or apoptosis, fully retains the ability to suppress SLC7A11 and trigger ferroptosis under oxidative stress (Le et al., 2015).

The reasons for variable expression of CHAC1 in different cancers are still unclear, but it appears to be critically dependent on TP53 status. In tumors with frequent TP53 mutations, which confer ferroptosis resistance, CHAC1 levels are often elevated. Here, CHAC1 provides advantages by degrading GSH, releasing amino acids that fuel growth and enhancing glycolysis (Mehta et al., 2022). Together, these actions promote tumor proliferation and metastasis. These findings suggest that exploiting CHAC1 is potentially safe for TP53-mutant tumors. Interestingly, the elevated CHAC1 levels in TP53-mutant cells might represent an adaptive response, potentially compensating for defective ferroptosis pathways or preventing harmful GSH accumulation. Crucially, the ROS generated during GSH degradation by CHAC1 may also cause further TP53 mutations, creating a vicious self-reinforcing cycle: CHAC1 activity promotes the very TP53 mutations that allow the tumor to exploit CHAC1′s harmful effect for growth. Thus, a mutation in the *TP53* gene could confer protection against ferroptosis in a specific manner. Therefore, while the exact mechanisms controlling CHAC1 expression requires further study, a significant connection to the TP53 pathway is evident.

In cancer, the ability to trigger or prevent ferroptosis has far-reaching consequences for drug development and treatment resistance. Therefore, studies have investigated how CHAC1 is regulated in tumor cells and their microenvironment: Prostate cancer patients frequently develop resistance to docetaxel, a challenge in which cancer-associated fibroblasts (CAFs) have been shown to play a significant role through exosome-mediated signaling (Giraldo et al., 2019; Sun et al., 2018). As part of the tumor microenvironment, CAFs influence cancer progression and therapy response by secreting exosomes that carry regulatory molecules. CAFs can package miR-432-5p into exosomes and transfer them to nearby prostate cancer cells. In these cells, miR-432-5p binds to a site in the CHAC1 promoter, as identified by Zhao et al. (2024), leading to reduced CHAC1 expression and decreased GSH degradation. As a result, ferroptosis is inhibited, which contributes to increased resistance to docetaxel. These findings highlight the critical role of stromal-tumor interactions and suggest that targeting miR-432-5p or restoring CHAC1 function could represent promising strategies to overcome chemotherapy resistance in prostate cancer (Zhao et al., 2024).

In gastric cancer, the RNA demethylase ALKBH5 (Alkylated DNA repair protein alkB homolog 5) removes an m^6^A modification from CHAC1 mRNA, a mark normally associated with transcript stability. This demethylation leads to destabilization of the mRNA, resulting in reduced CHAC1 protein levels and inhibition of ferroptosis. In this way, ALKBH5 directly downregulates CHAC1 and contributes to ferroptosis resistance in gastric cancer cells (Chen et al., 2023).

#### 2.3.2 CHAC1-mediated ferroptosis in cancer and neurological disorders: mechanisms and therapeutic implications

Generally, cancer cell lines show a high variety in terms of sensitivity toward ferroptosis (Yang et al., 2014). In order to continue tumor growth, oncogenes or carcinogenic signaling contribute to avoid ferroptosis and thus also allow resistance toward treatments (Hirschhorn and Stockwell, 2019). The toxic microenvironment that cancer cells are constantly exposed to confronts them with oxygen limitation and nutrient deprivation resulting in a high metabolic demand (Cubillos-Ruiz et al., 2017). The additional high ROS levels make cancer cells very vulnerable toward ferroptosis leading to a high dependence on various defense mechanisms to detoxify lipid radicals or prevent cysteine starvation (Lei et al., 2022). Thus, cancer cells are under constant ER stress and to deal with these circumstances induce UPR pathways. Notably, the PERK-ATF4 arm of the UPR can be activated as an adaptive response to negatively regulate ferroptosis and promote survival. In this protective role, ATF4 drives the expression of the chaperone heat shock protein family A (Hsp70) member 5 (HSPA5), which in turn stabilizes and enhances the activity of GPX4, thereby inhibiting ferroptotic cell death (Chen et al., 2019).

In ferroptosis resistant melanoma cells, *CHAC1* was shown to be upregulated by NRF2 independent from ER stress signaling alongside the Aldo-Keto Reductase Family 1 Member C1 (AKR1C1) capable of degrading lipid radicals (Gagliardi et al., 2019). Despite the early upregulation of *CHAC1*, most melanoma cell lines appear to be resistant against ferroptosis. Further, susceptibility to ferroptosis appears to be connected to the differentiation of melanoma cells and cannot necessarily be applied to metastatic-derived cells. This difference may arise from the epithelial-mesenchymal transition (EMT)-associated gene expression reprogramming process. Interestingly, suppressing the ER stress pathway PERK-ATF4-CHOP did not affect CHAC1 expression during ferroptosis (Gagliardi et al., 2019). Although the exact role of CHAC1 in ferroptosis resistant cancer cells remains unclear, it presents an interesting target for cancer drug development as it plays a key role in multiple cellular processes associated with cancer.

As controlled induction of ferroptosis might prove a promising target for cancer research, various pharmacological substances have been tested for possibly inducing ferroptosis. Among these, artesunate, sevoflurane, dihydroartemisinin, brusatol, nisin, hederagenin, glaucocalyxin A, nootkatone, cannabinoids and ophiopogonin B successfully promote ferroptosis in diverse cancer cell types mediated by the ER stress branch ATF4-CHOP-CHAC1 (Besser et al., 2024; Joo et al., 2012; Lu et al., 2024; Wang et al., 2019; Wang et al., 2021; Wang et al., 2022; Wang et al., 2025; Xu et al., 2022; Yu et al., 2024; Zhang et al., 2022).

In 2012, Joo et al. were the first to encounter CHAC1 expression in the context of cancer cell death (Joo et al., 2012). They showed that the *CHAC1* gene is highly upregulated in HNSC cancer after treatment with nisin which was then followed by cell death (Joo et al., 2012). Treatment with hederagenin in lung cancer cells, leads to a strong upregulation of the *CHAC1* gene even though basal CHAC1 expression is significantly lower than in normal tissues (Lu et al., 2024). Interestingly, artesunate-mediated induction of ferroptosis appears to be rather controversial or possibly cell type specific. In 2014, Dixon et al. found no *CHAC1* upregulation after exposing HT-1080 (fibrosarcoma cell line) and Calu-1 (non-small-cell lung cancer) cells to artesunate, whereas in more recent studies, a significant increase in CHAC1 expression in Burkitt’s lymphoma cells was observed (Dixon et al., 2014; Wang et al., 2019). Tumor growth inhibitor glaucocalyxin A induces the mitochondrial apoptosis pathway. Here, *CHAC1* is upregulated by the ATF4-CHOP cascade and leads to cell death by perturbation of the redox homeostasis through changes in the ROS levels (Wang et al., 2022).

The pathological significance of CHAC1 and ferroptosis extends beyond cancer, having also been established in various neurological disorders. Among these are neurodegenerative diseases, which are characterized by progressive neuronal impairment or loss leading to cognitive and motor decline (Wu et al., 2018). Inhibitors of ferroptosis have already proven to have a protective effect on degenerative brain disorders such as Parkinson’s, Huntington’s, and Alzheimer’s diseases, as well as in traumatic and hemorrhagic brain injury (Chen et al., 2015; Do Van et al., 2016; Gascón et al., 2016; Guiney et al., 2017; Hambright et al., 2017; Li et al., 2017; Skouta et al., 2014; Zille et al., 2017).

Although direct evidence of CHAC1 involvement in human neurodegeneration remains limited, its well-established role in GSH catabolism positions it mechanistically at the intersection of ferroptotic pathways. Pharmacological targeting of CHAC1-mediated processes therefore represents a rational therapeutic strategy, potentially disrupting the cycle of GSH depletion, ER stress amplification and iron-dependent peroxidation that drives neuronal loss.

### 2.4 CHAC1-mediated regulation of stem cell fate and development *via* NOTCH1 signaling

Given the central role of glutathione in maintaining redox balance, its levels are a key determinant of stem cell fate. Pluripotent stem cells are known to have high GSH levels under reduced conditions. However, when these cells differentiate, GSH decreases nearly four times and oxidative conditions increase (Dannenmann et al., 2015; Zhou et al., 2016). These shifts in the redox state can modulate key signaling pathways that govern cell fate, such as NOTCH (Neurogenic locus notch homolog protein).

**FIGURE 6:**
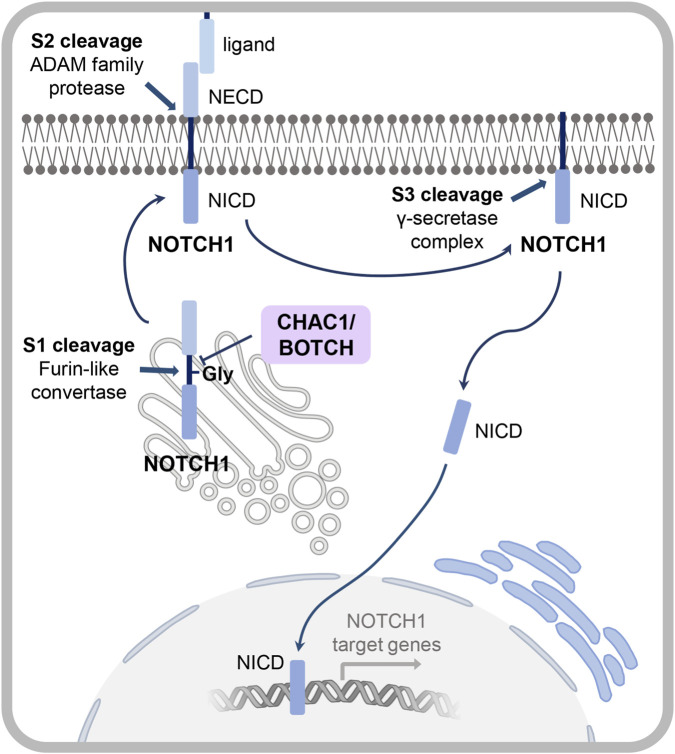
CHAC1 mediated regulation of NOTCH1 signaling. CHAC1 negatively regulates NOTCH1 activity by inhibiting the initial S1 furin-like cleavage step of NOTCH1 maturation in the Golgi apparatus. CHAC1/BOTCH mediates the deglycination of a glutamate residue by replacing it with 5-oxoproline. This prevents subsequent S2 and S3 maturation steps mediated by the ADAM family protease and the γ-secretase complex at the cellular membrane resulting in an inhibition of the transcription of genes downstream of the NOTCH1 signaling pathway. NICD, NOTCH intracellular domain; NECD, NOTCH extracellular domain.

The NOTCH signaling pathway is highly conserved across metazoans, with homologs identified in numerous species that share similar structures and core signaling components (Coffman et al., 1990; Stubbs et al., 1990; Yochem et al., 1988). This pathway is a fundamental regulator of cellular processes such as cell fate decisions, embryonic development, tissue repair and cancer (Siebel and Lendahl, 2017). During maturation, the NOTCH precursor undergoes a complex process of modifications and cleavages, like the S1 cleavage catalyzed by a furin-like protease before translocation of the mature protein to the cell membrane (Kopan and Ilagan, 2009). The enzyme CHAC1 (CHAC1/BOTCH for “blocking NOTCH”) is a key regulator of this process (Figure 6). It inhibits NOTCH1 signaling by using its γGCT activity to cleave a γ-glutamyl-cysteine bond at glutamate^1669^ of the immature NOTCH receptor, thereby preventing the essential S1 cleavage and subsequent activation (Chi et al., 2014).

The physiological importance of this mechanism is underscored by severe developmental phenotypes upon its disruption. In mice, CHAC1/BOTCH promotes neuronal differentiation by inhibiting NOTCH1 and both its depletion and overproduction severely disrupt neurogenesis (Chi et al., 2012). Similarly, knockdown of *CHAC1/BOTCH* in zebrafish leads to severe defects in brain, heart and myotome development, resulting in embryonic lethality, highlighting its conserved and essential role (Yadav et al., 2019). Interestingly, while this study discovered that CHAC1/BOTCH-mediated regulation of GSH levels controls development, other studies suggest its developmental role is primarily based on the inhibition of NOTCH1 signaling (Chi et al., 2012; Yadav et al., 2019). Through its interaction with NOTCH1, CHAC1/BOTCH inhibits NOTCH1 activity and thereby prevents neuronal stem cell self-renewal (Chi et al., 2012). Mitochondrial dynamics also play a central role in stem cell fate decisions. ROS-induced mitochondrial fragmentation, a process occurring during aging and in neurodegenerative diseases, leads to a loss of mitochondrial function known to enhance pathological development (Burté et al., 2015; Itoh et al., 2013; Scheckhuber et al., 2007). This mitochondrial fission triggers an upregulation of *CHAC1/BOTCH* through the transcription factor NRF2 and ROS signaling, which in turn inhibits stem cell self-renewal and promotes cell differentiation (Khacho et al., 2016). Consequently, a decrease in CHAC1/BOTCH expression levels, caused by the loss of NRF2, rescues NOTCH1 signaling and restores neuronal stem cell self-renewal (Khacho et al., 2016).

#### 2.4.1 The antagonistic roles of CHAC1 and CHAC2 in stem cell regulation

The function of CHAC1 is further refined by its competitive interaction with its homolog, CHAC2. Intriguingly, a competitive relationship between the homologues CHAC1 and CHAC2 was observed in human embryonic stem cells (hESCs), where they play opposing roles in maintaining cellular GSH levels and regulating differentiation (Wang et al., 2017). In contrast to CHAC1, which promotes stem cell differentiation, CHAC2 is essential for maintaining self-renewal capacity. CHAC2 supports the expression of pluripotency genes through its impact on GSH homeostasis. Furthermore, CHAC2 appears to negatively influence CHAC1-mediated GSH degradation, suggesting a direct competitive interaction (Wang et al., 2017). The functional importance of this antagonism is demonstrated by the finding that the loss of self-renewal caused by CHAC2 knockdown is completely rescued by the additional knockdown of CHAC1. This indicates that the primary role of CHAC2 in pluripotency is to restrain the differentiation activity of CHAC1 (Khacho et al., 2016; Wang et al., 2017).

#### 2.4.2 CHAC1 in neuroprotection and organ injury

Similar to its role in stem cell fate, CHAC1/BOTCH exhibits a dual function in regulating neuroprotection and inflammation following neuronal damage and organ injury. Through its ability to block NOTCH1 maturation, CHAC1/BOTCH was identified as Neuroprotective Gene 7 (NPG7) due to its protective effects in neuronal contexts. Specifically, CHAC1 provides neuroprotection during damage induced by subarachnoid or intracerebral hemorrhage (Chen et al., 2021; Liu, W. et al., 2019; Mei et al., 2017). For instance, treatment of subarachnoid hemorrhage with bone marrow mesenchymal stem cells resulted in upregulation of *CHAC1/BOTCH*, which significantly reduced neuroinflammation by blocking NOTCH1 signaling (Li et al., 2019; Liu, W. et al., 2019). Overexpression of CHAC1/BOTCH also demonstrated notable rescue effects against neuronal injury, highlighting its potential as a therapeutic target in stroke and neuroinflammation research (Mei et al., 2017). Conversely, downregulation of CHAC1/BOTCH was associated with increased neuronal damage and impaired recovery (Chen et al., 2021).

The regulation of CHAC1/BOTCH involves additional molecular players: during intracerebral hemorrhage, inhibition of CHAC1/BOTCH by the androgen receptor (AR) and Jumonji-domain containing protein-3 (JMJD3) led to elevated NOTCH1 levels and exacerbated neuroinflammation (Chen et al., 2021). In glaucoma, a condition involving retinal ganglion cell death due to axonal injury, CHAC1 expression is induced by ER stress signaling. Among stress-responsive genes, *ATF4* was most prominently upregulated, though other related genes were also significantly elevated (Yasuda et al., 2014). Furthermore, in a lipopolysaccharide-induced inflammatory model, propofol treatment inhibited ferroptosis after brain injury. This was evidenced by reduced levels of ferroptosis markers such as MDA and iron, along with repressed CHAC1 expression (Zhou, Y. et al., 2024).

In contrast to its neuroprotective role, CHAC1 promotes organ injury in other contexts primarily through ferroptosis regulation. Upregulation of *CHAC1* has been consistently observed in models of retinal damage, kidney and liver injury and heat shock–induced oxidative stress, often in tandem with ER stress (Chen et al., 2024; Cheng et al., 2024; Cui et al., 2021; Ju et al., 2025; Kolligundla et al., 2025; Liu et al., 2023).

In acetaminophen (APAP)-induced liver injury, CHAC1 indirectly promotes ferroptosis by modulating the ferroptosis suppressor ARF6. CHAC1-mediated GSH degradation reduces S-glutathionylation of ARF6 at cysteine 90, thereby inactivating ARF6 and enhancing ferroptotic cell death (Ju et al., 2025). This is consistent with the known role of ARF6 as a negative regulator of ferroptosis in cancers such as pancreatic and gastric carcinoma (Geng and Wu, 2022).

Similarly, *CHAC1* is upregulated in various kidney disease models (Kolligundla et al., 2025; Ying et al., 2025). In renal tubular epithelial cells treated with calcium oxalate (CaOx) to induce kidney stone formation, CHAC1 expression increases alongside a decreased GSH level, reduced GPX4 activity, increased iron and MDA content and elevated ROS and lipid peroxidation. Intriguingly, Ying et al. also found an upregulation of the autophagy marker protein LC3 downstream of CHAC1, suggesting an autophagy-mediated induction of ferroptosis (Ying et al., 2025). Another recent study showed increased calcium accumulation in renal tubular epithelial cells in response to CHAC1 overexpression, which was ameliorated by CHAC1 knockdown (Dong et al., 2025). In kidney disease, *CHAC1* upregulation was induced by cysteine and methionine deprivation, treatment with erastin and exposure to tunicamycin. Additionally, Kolligundla et al. presented a heterozygous CHAC1 deletion mouse model that was protected from kidney disease progression across three different disease models, without any known phenotypic changes under control conditions (Kolligundla et al., 2025).

Despite its injury-promoting effects in some organs, CHAC1 contributes to tissue repair in certain contexts. In chicken myoblasts, *CHAC1* is upregulated during proliferation, differentiation and muscle regeneration following injury (Chen et al., 2024). This expression is finely tuned by a post-transcriptional regulatory axis wherein miR-301a-5p suppresses *CHAC1*, while the long non-coding RNA lncMDP1 buffers this inhibition, facilitating muscle recovery (Chen et al., 2024).

### 2.5 CHAC1 in inflammatory and infectious disease context

Generally, the inflammatory response is strongly related to the induction of ferroptosis. The production of MDA and ROS during ferroptosis triggers significant oxidative stress and inflammation (Yuan et al., 2024). This process leads to the release of cellular cytokines known as damage-associated molecular patterns (DAMPs), which promote a proinflammatory state (Conrad et al., 2016). In sepsis, CHAC1 upregulation is detrimental, as it promotes ferroptosis and impairs the immune response in dendritic cells. This is evidenced by the finding that the protective enzyme Sestrin2 (Sesn2) inhibits ferroptosis by downregulating the ATF4-CHOP-CHAC1 signaling pathway (Li, J.-Y. et al., 2021). In periodontitis, CHAC1 downregulation is observed in LPS-treated gingival fibroblasts, suggesting a potential protective role of CHAC1 loss in this specific inflammatory context (Yuan et al., 2024).

Furthermore, its role in the immune response is exemplified in cystic fibrosis. Cystic fibrosis is characterized by Cystic Fibrosis Transmembrane Conductance Regulator (CFTR) dysfunction in the airway epithelium, leading to impaired mucociliary clearance and defective innate immune responses which promote chronic bacterial colonization. In this disease, the failure to upregulate CHAC1 is detrimental. Here, airway epithelial cells cannot induce CHAC1 expression in response to bacterial or viral infection. This deficiency contributes to the excessive inflammatory response typical of the disease (Cohen and Prince, 2012; Perra et al., 2018). Whereas non-cystic fibrosis cells upregulate *CHAC1* following infection with various pathogens (including *Pseudomonas aeruginosa*, human coronavirus, cytomegalovirus, tick-borne flaviviruses, Zika virus, and *Mycoplasma hominis*), cystic fibrosis cells show an impaired response. These cells not only fail to induce CHAC1 expression but also exhibit significantly reduced ATF4 levels (Perra et al., 2018).

## 3 Mammalian CHAC2: defining its role in glutathione homeostasis, cancer progression and stem cell fate

In addition to CHAC1, which is found exclusively in higher eukaryotes, organisms from bacteria to mammals possess a CHAC2 enzyme that exhibits a highly similar glutathione-specific γGCT activity (Kaur et al., 2017). Early studies indicated that CHAC2 expression was not differentially regulated and was generally higher than that of CHAC1, suggesting a role in the basal turnover of GSH rather than the stress- and signaling-associated functions of CHAC1. This interpretation was further supported by CHAC2’s lower catalytic activity compared to CHAC1 (Kaur et al., 2017).

However, more recent research has revealed that CHAC2 expression is subject to complex regulation and is implicated in diverse cellular processes. Its expression is significantly increased in lung adenocarcinoma, downregulated in gastric and colorectal cancers and altered during the differentiation of pluripotent embryonic stem cells (Liu et al., 2017; Peng et al., 2023; Wang et al., 2017). Furthermore, *CHAC2* was upregulated in a model of APAP-induced hepatic damage following treatment with naringin (Zhai et al., 2022). These findings implicate CHAC2 in various cellular functions, including DNA replication and repair, cell cycle regulation, apoptosis, and stem cell differentiation, indicating that its functional repertoire is more versatile than initially presumed (Chand et al., 2022; Peng et al., 2023; Wang et al., 2017). Interestingly and in contrast to CHAC1, CHAC2 is not localized to the mitochondria (Ward et al., 2024). This difference in subcellular localization, combined with its distinct expression patterns, suggests that the two proteins play different roles in regulating cellular GSH levels (Ward et al., 2024).

### 3.1 CHAC2 and cancer

In contrast to CHAC1, the general role of CHAC2 in cancer and programmed cell death remains elusive. This enzyme appears to have context-dependent functions across different cancer types. Primarily, CHAC2 can inhibit tumor progression by decreasing cellular GSH levels and inducing mitochondrial apoptosis (Liu et al., 2017). Similar to *CHAC1*, *CHAC2* expression varies significantly between tissues. It is downregulated in gastric and colorectal cancers but upregulated in lung adenocarcinoma and breast tumors compared to normal tissue (Chand et al., 2022; 2022; Liu et al., 2017; Peng et al., 2023). Interestingly, this differential expression correlates with opposing patient outcomes. High CHAC2 levels are associated with a higher 3-year survival rate in gastric and colorectal cancer patients. Conversely, in lung and breast cancer, elevated CHAC2 correlates with advanced tumor stage and grade, indicating a worse prognosis (Chand et al., 2022; 2022; Liu et al., 2017; Peng et al., 2023).

In gastric and colorectal cancer, high CHAC2 expression leads to reduced glutathione, elevated intracellular Ca^2+^ and increased ROS, resulting in inhibited tumor growth, proliferation and migration. This is accompanied by the upregulation of ER stress-related proteins, including PERK, IRE1, ATF4, CHOP, XBP-1 and ATF6. CHAC2 itself is regulated both transcriptionally and post-translationally, with its degradation mediated by the ubiquitin-proteasome pathway (Liu et al., 2017).

In lung cancer, however, the CHAC2-mediated increase in ROS triggers the MAPK pathway. In this context, CHAC2 expression promotes cancer growth, inhibits apoptosis and accelerates cell cycle progression through MAPK hyperactivation (Peng et al., 2023).

Furthermore, in breast cancer, Chand et al. observed a correlation between high CHAC2 levels and mutant TP53, potentially due to hypomethylation of the *CHAC2* promoter. CHAC2 expression is also closely linked to several genetic mutations prevalent in breast cancer, most commonly TP53, as well as CDH1, BIRC6, DYNC2H1 and UTRN (Chand et al., 2022).

Thus, like CHAC1, CHAC2 represents an intriguing and complex target for further cancer research.

### 3.2 CHAC2 in development and stem cell fate decision

A role for CHAC2 in stem cell fate is likely, given the established importance of GSH homeostasis in differentiation and the known function of its homolog, CHAC1. A high GSH concentration and a reduced cellular state are known to promote self-renewal, allowing the GSH-to-ROS ratio to be a key determinant of cell fate (Dannenmann et al., 2015; Ji et al., 2010; Zhou et al., 2016).

In human embryonic stem cells, CHAC2 is highly enriched and is crucial for maintaining self-renewal capacity. It achieves this by controlling the expression of pluripotency genes through the regulation of GSH homeostasis and the suppression of the mesoderm-inducing protein Brachyury (Wang et al., 2017). Furthermore, CHAC2 competes with CHAC1 to influence GSH homeostasis and, consequently, stem cell fate decisions. Despite having a significantly lower catalytic activity, high levels of CHAC2 negatively regulate CHAC1 enzyme activity. This interplay may serve as a sophisticated feedback mechanism to fine-tune GSH levels and prevent their excessive depletion. Furthermore, CHAC2 also maintains pluripotency through a CHAC1-independent pathway by regulating the NRF2-GCL axis, thereby sustaining GSH biosynthesis and concurrently suppressing ROS production (Wang et al., 2017).

The significance of CHAC2-NRF2 signaling pathway extends beyond development. For instance, in a model of APAP-induced liver injury, the protective antioxidant and anti-inflammatory effects of the bioflavonoid naringin are highly dependent on the upregulation of both *CHAC2* and *NRF2* (Zhai et al., 2022). This demonstrates the critical role of the CHAC2-NRF2 signaling pathway in protecting against oxidative stress and highlights CHAC2 as a potential therapeutic target for mitigating APAP-induced liver damage (Zhai et al., 2022).

**FIGURE 7:**
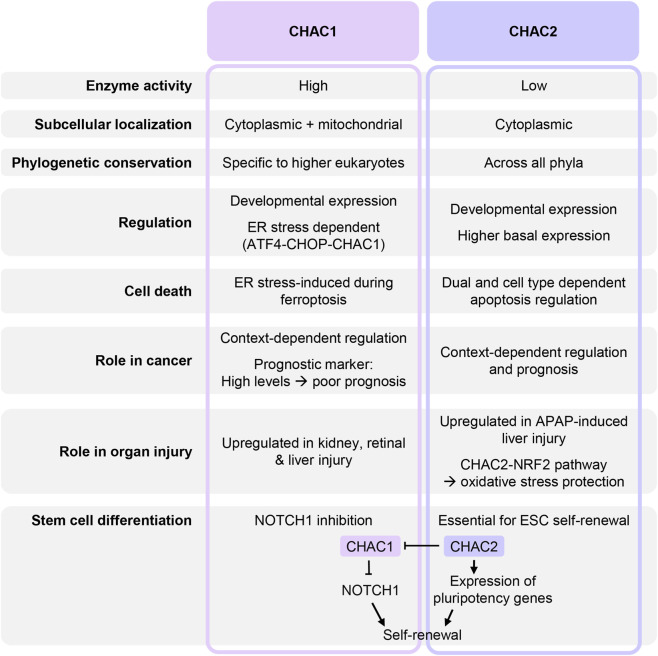
Comparative overview of mammalian CHAC1 and CHAC2. Schematic summarizing the key structural, regulatory and functional distinctions between the two enzymes.

Taken together, the literature indicates that while both CHAC1 and CHAC2 participate in related biological processes, their different structural and regulatory properties suggest complementary rather than redundant functions. As shown in Figure 7, these related enzymes show fundamental differences in their expression patterns, physiological roles and disease implications across multiple biological contexts.

## 4 CHAC enzymes beyond mammals

Research on ChaC-like proteins has primarily focused on the mammalian homologs CHAC1 and CHAC2, with very few studies characterizing these enzymes in other organisms. As CHAC1 is found predominantly in higher eukaryotes, most other species possess only the CHAC2 homolog.

The initial identification of a *chaC*-like gene occurred in the *E. coli cha* operon (Ivey et al., 1993). This operon comprises three genes: *chaA*, *chaB*, and *chaC*. While *chaA* encodes a characterized Na^+^(Ca^2+^)/H^+^ antiporter, *chaB* and *chaC* were originally identified as putative, though uncharacterized, regulators of *chaA* (Oshima et al., 1996). It is from this role in cation transport regulation that the ChaC-like enzyme family derives its alternative name: cation transport regulator-like proteins (Radchenko et al., 2006).

The soil-borne plant pathogen *R. solanacearum* employs a sophisticated virulence strategy through its type III secretion effector RipAY. This effector contains a ChaC-like domain and exhibits γGCT activity, thereby targeting the host´s GSH-based defense system (Wei et al., 2017). Interestingly, RipAY’s enzymatic activity requires the activation by eukaryotic thioredoxins, which are highly abundant in plants during pathogen infection (Tada et al., 2008). Furthermore, the degradation of GSH and the suppression of immune responses requires phosphorylation of RipAY (Wei et al., 2017).

Beyond bacteria, ChaC-like proteins play roles in diverse pathogens. For example, in the protozoan parasite *L. major*, two CHAC2 enzymes, CHAC2a and CHAC2b, have been identified. CHAC2a is constitutively expressed, whereas CHAC2b exhibits higher catalytic activity and is induced during sulfur starvation, suggesting a specialized role in nutrient stress response (Das et al., 2022).

### 4.1 CHAC enzymes in plants

In plants, GSH plays a vital role in mediating biotic stress (Dubreuil-Maurizi and Poinssot, 2012). Although its precise function within the complex signaling networks of plant immunity is not fully elucidated, a connection to the NPR1 (NPR1 or NIM1)-dependent salicylic acid (SA)-mediated pathway is theorized (Kumar et al., 2010; Lu et al., 2023).

Consequently, the current understanding of glutathione-degrading enzymes in plants remains limited. The ChaC-like family, in particular, is relatively unexplored, with the few existing studies primarily focused on *Arabidopsis thaliana*, rice (*Oryza sativa*), and wheat (*Triticum aestivum* L.) (Kumar et al., 2015; Qi et al., 2005; Zhang, L. et al., 2024). The initial identification of ChaC-like enzymes in plants occurred in a 2005 study on submergence-induced genes in rice, where the upregulated gene *OsCTP* was noted for its high similarity to the *E. coli* cation transport protein ChaC (Qi et al., 2005).

The cytosolic degradation of GSH in plants is primarily facilitated by two enzyme types: GGCT and GGP (γ-glutamylpeptidase) (Ito and Ohkama-Ohtsu, 2023). The GGCT family is subdivided into three groups: plant-specific GGCT (psGGCT), γ-glutamylamine cyclotransferases (GGACT), and the ChaC-like family proteins (Kumar et al., 2015). These groups exhibit distinct substrate specificities; ChaC-like enzymes act specifically on GSH, GGACT enzymes act on γ-glutamylamine amino acids, and GGCTs have a broader activity towards γ-glutamyl amino acids in general. Despite low sequence similarity, all these proteins share a common BtrG-fold, conferring a high degree of structural conservation (Kumar et al., 2015).

In *Arabidopsis*, the ChaC-like family comprises three members: GGCT2; 1, GGCT2; 2, and GGCT2; 3, which are considered functional homologues of mammalian CHAC2 (Kaur et al., 2017; Kumar et al., 2015; Paulose et al., 2013). Similar to their mammalian homologue, these enzymes specifically degrade intracellular GSH while yielding 5-oxoproline and cysteinylglycine (Kumar et al., 2015; Paulose et al., 2013). Beyond the ChaC-like family, the *Arabidopsis* genome encodes two GGACT members and nine psGGCT proteins (Kumar et al., 2015). The primary physiological roles of GGCTs are associated with plant growth, development and responses to various stresses, such as drought, consistent with their expression in roots, stems and developing grains (Zhang, L. et al., 2024).

The individual *Arabidopsis* CHAC homologues appear to have divergent functions. GGCT2; 1 exhibits an expression pattern and functional profile more analogous to mammalian CHAC1, suggesting a role in amino acid metabolism or signaling pathways. This enzyme confers significant tolerance to salt, drought, and cold stress in roots. Its promoter region contains eight stress-responsive cis-acting elements linked to phytohormones and stress pathways, including abscisic acid, MeJA (methyl jasmonate), gibberellin and salicylic acid (Ghosh et al., 2022). Overexpression of GGCT2; 1 enhances resistance to As^III^ stress and reduces levels of the lipid peroxidation marker MDA, highlighting its potential as a target for crop improvement (Singh et al., 2023). In contrast, the other two enzymes GGCT2; 2 and GGCT2; 3 were initially assigned a role in maintaining GSH homeostasis (Bachhawat and Kaur, 2017). However, a more recent hypothesis proposes that GGCT2; 2 promotes GSH accumulation in pluripotent cells during stem cell fate decisions in *Arabidopsis*, mirroring the function of mammalian CHAC2 (Ito and Ohkama-Ohtsu, 2023). This is supported by its strong upregulation in immature organs like root tips, where GSH is required for cell division in the meristem (Ito et al., 2022; Vernoux et al., 2000). The promoter of *AtGGCT2;2* contains cis-elements responsive to auxin, MeJA and low temperature. Furthermore, unlike GGCT2; 1, GGCT2; 2 expression is highly upregulated by exogenous GSH supplementation indicating a substrate-dependent regulatory mechanism (Ghosh et al., 2022).

Given the growing body of literature on CHAC proteins, a comprehensive overview of all published work is beyond the scope of this review. Therefore, the following supplemental tables present key studies that highlight the major roles of CHAC proteins (Supplementary Tables S1–S6).

## 5 Conclusion

The family of the ChaC-like enzymes has attracted increasing attention over the past few years. These enzymes are highly conserved across all phyla and are involved in diverse processes such as regulating oxidative stress, neurogenesis, cell death and stem cell fate decisions. Pathogenic bacteria utilize CHAC enzymes, while in plants, they help respond to environmental stressors and influence growth, development and pluripotency.

In mammals, CHAC1 plays a key role in cell cycle regulation and the maintenance of redox balance and is centrally involved in ferroptosis, stem cell differentiation and development. It has also been linked to various diseases, including cancer, neurodegenerative disorders and inflammation.

Specifically in oncology, *CHAC1* shows varying expression patterns across different cancer types, where it can promote ferroptosis and increase oxidative damage. The reason for this variation is still unknown but may be associated with mutations in the tumor suppressor gene *TP53*.

The effect of CHAC1 overexpression on cellular susceptibility to chemotherapy highlights its potential as a drug target. As an inducer of ferroptosis, inhibiting CHAC1-induced cell death may also hold therapeutic relevance for conditions like neurodegenerative diseases.

In contrast to CHAC1, less is known about its homologue, CHAC2. Current evidence indicates that CHAC2 helps maintain GSH homeostasis and may also play a role in cancer progression and development. During development, CHAC2 exhibits a role complementary to CHAC1 by enhancing pluripotency and stem cell renewal, whereas CHAC1 promotes differentiation. Despite these insights, the distinct physiological roles of these two enzymes are still not fully understood. While research interest in CHAC1 is growing, CHAC2 remains significantly understudied.

Moving forward, it is critical to recognize that the current research focus on CHAC enzymes disproportionately targets their stress-induced roles, leaving their fundamental biology poorly understood. Critical knowledge gaps persist in both unresolved basic enzymology and the comprehensive understanding of their multi-level regulation. The mechanisms controlling their transcription, post-transcriptional mRNA fate and post-translational modifications remain largely uncharacterized. Furthermore, the physiological purpose of constitutive GSH degradation and the systemic consequences of genetic knockout are barely known. This reliance on correlative evidence from disease contexts has reduced CHAC enzymes to mere stress markers and overlooked their potential as dynamically regulated metabolic integrators. An advanced comprehension of the basic biology of CHAC enzymes, derived from rigorous biochemical and genetic studies, is therefore a prerequisite for their rational therapeutic exploitation.
