# Supplementary material for: The strategic breakdown: CHAC enzymes as regulators of glutathione homeostasis and disease implications
Source: Front Mol Biosci. 2025 Dec 18;12:1724944. doi: 10.3389/fmolb.2025.1724944 (PMC12756093; doi:10.3389/fmolb.2025.1724944)
Supplement: Supplementary file 1 [file Supplementaryfile1.docx]

Supplemental table S1: CHAC1 expression levels across different cancer types.

| **Cancer type** | **CHAC1 (Tumor vs. Normal)** | **Changes in CHAC1 expression** | **TP53 status** | **Ferroptosis susceptibility** | **Literature** |
| --- | --- | --- | --- | --- | --- |
| **Carcinomas** | | | | | |
| Gastric cancer | **-** | ↑  via *H. pylori* infection | CHAC1 induces *TP53* mutations | - | Wada et al., 2018 |
| Colon adenocarcinoma (COAD) | **↑** | - | - | - | Li et al., 2021 |
| Rectum adenocarcinoma (READ) | **↑** | - | - | - | Li et al., 2021 |
| Gastric cancer | **↓** | ↓  via ophiopogonin B | - | - | Zhang et al., 2022 |
| Breast cancer | **↑** | - | Mutant *TP53* tumor sets | - | Goebel et al., 2012; Mehta et al., 2022 |
| Ovarian cancer | **↑** | - | - | - | Goebel et al., 2012 |
| Triple negative breast cancer | **-** | ↑  after cysteine deprivation | - | susceptible to ferroptosis | Chen et al., 2017 |
| Breast invasive carcinoma (BRCA) | **↑** | - | - | - | Li et al., 2021 |
| Uterine corpus endometrial carcinoma (UCEC) | **↑** | - | - | - | Li et al., 2021 |
| Head and neck squamous cell carcinoma (HNSC) | **↓** | ↑  via nisin | - | susceptible to apoptosis | Joo et al., 2012;  Li et al., 2021 |
| Thyroid carcinoma (THCA) | **↑** | - | - | - | Li et al., 2021 |
| Oral squamous cell carcinoma | **↓** | ↑  via glaucocalyxin A | - | susceptible to ferroptosis | Wang et al., 2022 |
| Hepatocarcinoma | **-** | ↑  after serine depletion | - | - | Hamano et al., 2020 |
| Cholangio carcinoma (CHOL) | **↑** | - | - | - | Li et al., 2021 |
| Liver hepatocellular carcinoma (LIHC) | **↑** | - | - | - | Li et al., 2021 |
| Primary liver cancer | **-** | ↑  via dihydroartemisinin | PLC cell lines with varying p53 status | susceptible to ferroptosis | Wang et al., 2021 |
| Lung squamous cell carcinoma (LUSC) | **↑** | - | - | - | Li et al., 2021 |
| Lung adenocarcinoma (LUAD) | **↑** | - | - | - | Li et al., 2021;  Pan et al., 2024 |
| Lung cancer | **↓** | ↑  via hederagenin | - | susceptible to ferroptosis | Lu et al., 2024 |
| Non-small cell lung cancer | **-** | ↑  under cysteine starvation | - | susceptible to ferroptosis | Ward et al., 2024 |
| Bladder urothelial carcinoma (BLCA) | **↑** | - | - | - | Li et al., 2021 |
| Kidney renal clear cell carcinoma | ↓* | - | - | - | Li et al., 2021 |
| Kidney renal papillary cell carcinoma (KIRP) | **↓** | - | - | - | Li et al., 2021 |
| Bladder cancer | **-** | ↑  via brusatol | - | susceptible to ferroptosis | Yu et al., 2024 |
| Prostate cancer | **-** | - | - | - | Zhao et al., 2024 |
| **Gliomas** | | | | | |
| Brain lower grade glioma (LGG) | **↓** | - | - | - | Li et al., 2021 |
| Glioma | **-** | ↑  via sevoflurane | - | susceptible to ferroptosis | Xu et al., 2022 |
| Glioblastoma multiforme | **-** | ↑  via nootkatone | - | susceptible to ferroptosis | Wang et al., 2025 |
| **Hematologic Malignancies** | | | | | |
| Burkitt’s lymphoma | **-** | ↑  via artesunate | - | susceptible to ferroptosis | Wang et al., 2019 |
| Lymphoblastic leukemia | **-** | ↑  via cannabinoids | - | susceptible to apoptosis | Besser et al., 2024 |
| **Melanomas** | | | | | |
| Metastatic melanoma | **↑** | - | - | ferroptosis resistant | Gagliardi et al., 2019 |
| Uveal melanoma | **↑** | - | - | - | Liu et al., 2019 |

*higher in samples with higher malignancy and later stages

Supplemental table S2: A representative selection of key publications on CHAC1 characterization and regulation.

| **Content summary** | **Publication** |
| --- | --- |
| **CHAC1 characterization and enzymology** | |
| Identification and characterization of the GGCT family protein | Oakley et al., 2008 |
| Identification of CHAC1 as a pro-apoptotic component of the Unfolded Protein Response (UPR) downstream of the ATF4-ATF3-CHOP cascade | Mungrue et al., 2009 |
| Functional characterization of CHAC1 enzyme activity | Kumar et al., 2012 |
| *CHAC1* regulation via ATF4, ATF3 and CEBPβ and an ATF/CRE regulatory element | Crawford et al., 2015 |
| Review on glutathione degradation including CHAC1, CHAC2 and γGTs, presenting a new glutathione cycle | Bachhawat  and Kaur, 2017 (Review) |
| Review on the new glutathione cycle and intracellular glutathione (GSH) degradation | Bachhawat and Yadav, 2018 (Review) |
| Bioinformatic analysis of the CHAC1 active site and functional *in vivo* activity assays in yeast | Suyal et al., 2023 |
| Review on CHAC1 in ferroptosis and cancer | Sun et al., 2024 (Review) |
| **Transcriptional and post-transcriptional regulation** | |
| Identification of CHAC1 as proapoptotic component of the UPR downstream of the ATF4-ATF3-CHOP cascade | Mungrue et al., 2009 |
| During arsenite stress, CHAC1 protein expression is negatively regulated by TRIB3 | Örd et al., 2016 |
| Extracellular serine depletion induces transcriptional activation of *CHAC1* gene downstream of ATF4 | Hamano et al., 2020 |
| Regulation of the CHAC1 promoter region via ATF4, ATF3 and CHOP downstream of the UPR | Nomura et al., 2020 |
| DJ-1 negatively regulates ATF3 resulting in reduced CHAC1 levels in the context of Parkinson´s disease | Ge et al., 2022 |
| Regulation of CHAC1 via ATF-4 and the mTORC pathway in *C. elegans* | Statzer et al., 2022 |
| CHAC1 upregulated in response to arginine stress downstream the ATF4-CHOP axis | Miljkovic et al., 2023 |
| CHAC1 promotes apoptosis in HaCaT cells during arsenite stress by degrading GSH | Sumi et al., 2023 |
| ATF4 increases NRF2 transcription and induces CHAC1 levels, maintaining NRF2 activation | Kreß et al., 2023 |
| CHAC1 mRNA is upregulated in response to arsenite stress via inhibition of the m⁶A methyltransferase METTL3 | Qiu et al., 2025 |
| Exposure to the pollutant perfluorooctane sulfonate activates the ER stress-ATF4-CHAC1 axis leading to ferroptosis | Yang et al., 2025 |
| **CHAC1/BOTCH in development and signaling** | |
| First description of CHAC1/BOTCH as negative regulator of NOTCH1 | Chi et al., 2012 |
| CHAC1/BOTCH deglycinates NOTCH1 preventing S1 cleavage | Chi et al., 2014 |
| BOTCH plays a role in neuronal stem cell differentiation via NOTCH1 inhibition | Khacho et al., 2016 |
| CHAC2 inhibits CHAC1 function, regulating stem cell self-renewal | Wang et al., 2017 |
| CHAC1 required for Ca^2+^ signaling in zebrafish development | Yadav et al., 2019 |

Supplemental table S3: CHAC homologs in non-mammalian organisms.

| **Organism** | **Content** | **Publication** |
| --- | --- | --- |
| **Animals** | | |
| *Danio rerio* | CHAC1 required for Ca^2+^ signaling in zebrafish development | Yadav et al., 2019 |
| *Caenorhabditis elegans* | Regulation of CHAC1 via ATF-4 and the mTORC pathway in *C. elegans* | Statzer et al., 2022 |
| **Protozoans** | | |
| *Leishmania major* | CHAC2 important for *Leishmania* GSH homeostasis | Das et al., 2022 |
| **Plants** | | |
| Plants | Review on GSH degradation and GSH conjugates in plants | Ito and Ohkama-Ohtsu, 2023 (Review) |
| *Arabidopsis thaliana* | GGCT2;1 enzyme activity and upregulation under abiotic stress | Paulose et al., 2013 |
| *Arabidopsis thaliana* | Role of ChaC enzymes in *A. thaliana* | Kumar et al., 2015 |
| *Arabidopsis thaliana* | Comparative analysis of GGP1 function and GGCT2 in *Arabidopsis* | Ito et al., 2022 |
| *Arabidopsis thaliana Oryza sativa* | Description of GSH metabolizing genes under stress conditions in Arabidopsis and rice | Ghosh et al., 2022 |
| *Oryza sativa* | osCTP (similar to *E.coli* chaC) upregulated among submergence-induced genes in rice | Qi et al., 2005 |
| *Camelina sativa* | Overexpression of CsGGCT2;1 reduces arsenic toxicity and accumulation | Singh et al., 2023 |
| *Triticum aestivum L.* | Genome-wide identification, structure, evolution, and expression analysis of TaGGCT genes in wheat | Zhang et al., 2024 |
| **Prokaryotes** | | |
| *Escherichia coli* | Identification of *chaC* as a potential regulator of the *E. coli* *cha* (Ca^2+^/H^+^ antiporter) operon | Ivey et al., 1993 |
| *Ralstonia solanacearum Acidovorax citrulli* | Plant pathogen effector protein RipAY depletes host intracellular GSH dependent on thioredoxins | Fujiwara et al., 2016 |
| *Ralstonia solanacearum* | The plant pathogen effector protein RipAY is phosphorylated in plant cells | Wei et al., 2017 |

Supplemental table S4: Selected publications on CHAC2 function.

| **CHAC2-specific content** | **Publication** |
| --- | --- |
| *CHAC2* as a constitutively expressed gene regulating GSH homeostasis | Kaur et al., 2017 |
| CHAC2 inhibiting CHAC1 function, regulation of stem cell self-renewal | Wang et al., 2017 |
| CHAC2 as tumor suppressor is downregulated in gastric and colorectal cancers | Liu et al., 2017 |
| Structural and functional analysis of human CHAC2 | Nguyen et al., 2020 |
| Elevated CHAC2 levels correlate with poor outcome in breast cancer | Chand et al., 2022 |
| CHAC2 upregulation in response to naringin treatment of APAP-induced liver injury | Zhai et al., 2022 |
| CHAC2 promotes lung adenocarcinoma by ROS-mediated MAPK pathway activation | Peng et al., 2023 |
| CHAC2 expression not induced under cysteine starvation in non-small cell lung cancer | Ward et al., 2024 |
| Review on CHAC1 and CHAC2 in tumors | Zhang et al., 2024 (Review) |

Supplemental table S5: CHAC1 and CHAC2 in human disease.

| **Disease** | **CHAC1-related content** | **Publication** |
| --- | --- | --- |
| **Central Nervous System Disorders** | | |
| Intracerebral hemorrhage | BOTCH mediates neuroprotection by antagonizing NOTCH1 | Mei et al., 2017 |
| Cerebral ischemia-reperfusion injury | Botch exerts neuroprotective effects by antagonizing NOTCH1 signaling induced neuronal injury and inflammation | Li et al., 2019 |
| Early brain injury in subarachnoid hemorrhage | Role of BOTCH in early brain injury following subarachnoid hemorrhage by inhibition of NOTCH1-mediated neuroinflammation | Liu et al., 2019 |
| Intracranial hemorrhage | Androgen receptor promotes secondary brain injury via mediation of JMJD3-BOTCH-NOTCH1 axis | Chen et al., 2021 |
| **Kidney Disorders** | | |
| Kidney injury | Inhibition of ER stress-associated ferroptosis via ATF4-CHOP pathway, CHAC1 as ferroptosis marker | Cheng et al., 2024 |
| Kidney disease | Heterozygous CHAC1 deletion mouse model protected from kidney disease progression | Kolligundla et al., 2025 |
| Kidney stone formation | CHAC1 in ER stress-dependent ferroptosis in calcium oxalate kidney stone formation | Dong et al., 2025 |
| Kidney stone formation | Upregulation of CHAC1 and autophagy marker protein LC3 in calcium oxalate induced kidney stone formation | Ying et al., 2025 |
| **Liver and Metabolic Disorders** | | |
| Cystic fibrosis | Role of CHAC1 during Pseudomonas infection in cystic fibrosis | Perra et al., 2018 |
| APAP-induced liver injury | CHAC1-mediated GSH degradation reduces S-glutathionylation of ARF6, thereby inactivating ARF6 and enhancing ferroptotic cell death | Ju et al., 2025 |
| **Systemic Conditions** | | |
| Sepsis | Sesn2 downregulates the ATF4-CHOP-CHAC1 signaling pathway inhibiting ferroptosis induced by sepsis | Li et al., 2021 |
| Sepsis-induced brain injury | Propofol treatment represses ferroptosis accompanied by CHAC1 downregulation | Zhou, et al., 2024 |
| **Other Conditions** | | |
| Heat-exposure induced apoptosis | ER stress and oxidative stress depend on the ATF4-CHOP-CHAC1 pathway in heat exposure-induced apoptosis | Cui et al., 2021 |
| Retinal pigment epithelial cells with oxidative damage | Role of CHAC1 during oxidative stress-induced cell ferroptosis | Liu et al., 2023 |
| Muscle injury | Post-transcriptional inhibition of CHAC1 via miR-301a-5p | Chen et al., 2024 |
| Periodontitis | CHAC1 expression is downregulated in lipopolysaccharide-induced periodontitis cells | Yuan et al., 2024 |

Supplemental table S6: The roles of CHAC1 and CHAC2 in cancer.

| **Cancer type** | **CHAC1-specific content** | **Publication** |
| --- | --- | --- |
|  | Review on CHAC1 in ferroptosis  and cancer | Sun et al., 2024 (Review) |
|  | Review on CHAC1 and CHAC2 in tumors | Zhang et al., 2024 (Review) |
| **Carcinomas** | | |
| Gastric cancer | *H. pylori* infection upregulates CHAC1, leading to lower GSH and higher ROS, which can cause gene mutations | Wada et al., 2018 |
| Gastric cancer | CHAC1 levels are higher in GC cells than in normal tissue and increase in response to ophiopogonin B treatment | Zhang et al., 2022 |
| Gastric cancer | CHAC1 as target gene of the m^6^a demethyltransferase ALKBH5 in gastric cancer cells | Chen et al., 2023 |
| Kidney renal clear cell carcinoma | Sequencing analysis on CHAC1 regulation in KIRC and across various cancer types | Li et al., 2021 |
| Bladder cancer | Brusatol triggers ferroptosis via CHAC1-NRF2-SLC7A11 pathway in bladder cancer cells | Yu et al., 2024 |
| Prostate cancer | CHAC1 inhibited by miR-432-5p in cancer associated fibroblasts inhibiting ferroptosis | Zhao et al., 2024 |
| Breast and ovarian cancer | High CHAC1 expression as indicator for elevated risk of cancer malignancy | Goebel et al., 2012 |
| Triple negative breast cancer | Cysteine deprivation induced CHAC1 upregulation via GCN2-eIF2α-ATF4 axis | Chen et al., 2017 |
| Breast cancer | CHAC1 mRNA expression profiled across various breast cancer cells using various online tools | Mehta et al., 2022 |
| Head and neck squamous cell carcinoma | Nisin reduces HNSCC tumorigenesis via CHAC1 proapoptotic activity | Joo et al., 2012 |
| Oral squamous cell carcinoma | Glaucocalyxin A inhibits tumor growth via upregulation of the ATF4-CHOP-CHAC1 cascade | Wang et al., 2022 |
| Hepatocarcinoma | Extracellular serine depletion induces transcriptional activation of *CHAC1* gene downstream of ATF4 | Hamano et al., 2020 |
| Primary liver cancer | Dihydroartemisinin induces ferroptosis by CHAC1 upregulation via the ATF4-mediated UPR-branch | Wang et al., 2021 |
| Lung cancer | Hederagenin treatment leads to CHAC1 upregulation promoting lung cancer cell death | Lu et al., 2024 |
| Lung adenocarcinoma | E2F1-mediated CHAC1 upregulation promotes SUMOylation of PKM2 regulating energy metabolism in cancer cells | Pan et al., 2024 |
| Non-small cell lung cancer | ATF4-regulated mitoCHAC1 sustains function of Fe-S proteins under cysteine starvation | Ward et al., 2024 |
| **Gliomas** | | |
| Glioma | Sevoflurane induces ferroptosis via activation of ATF4-CHAC1 pathway | Xu et al., 2022 |
| Glioblastoma multiforme | Nootkatone inhibits the progression of GBM cells through activating the ATF4-CHOP-CHAC1 pathway | Wang et al., 2025 |
| **Hematologic Malignancies** | | |
| Burkitt’s lymphoma | Artesunate induces ferroptosis via ATF4-CHOP-CHAC1 pathway | Wang et al., 2019 |
| Lymphoblastic leukemia | Cannabinoid-mediated modulation of the NOTCH1 signaling pathway through ATF4-CHOP-CHAC1 leading to reduced tumor size and weight | Besser et al., 2024 |
| **Melanomas** | | |
| Metastatic melanoma | NRF2-dependent upregulation of CHAC1 in ferroptosis-resistant cells | Gagliardi et al., 2019 |
| Uveal melanoma | CHAC1 levels higher in UM than normal tissue correlating with poor prognosis | Liu et al., 2019 |
